# Mechanisms of Systemic Osteoporosis in Rheumatoid Arthritis

**DOI:** 10.3390/ijms23158740

**Published:** 2022-08-05

**Authors:** Peter Pietschmann, Maria Butylina, Katharina Kerschan-Schindl, Wolfgang Sipos

**Affiliations:** 1 Institute of Pathophysiology and Allergy Research, Center for Pathophysiology, Infectiology and Immunology, Medical University of Vienna, 1090 Vienna, Austria; 2 Department of Physical Medicine, Rehabilitation and Occupational Medicine, Medical University of Vienna, 1090 Vienna, Austria; 3 Clinical Department for Farm Animals, University of Veterinary Medicine Vienna, 1210 Vienna, Austria

**Keywords:** rheumatoid arthritis, osteoporosis, pathophysiology, fractures, bone mineral density, sarcopenia

## Abstract

Rheumatoid arthritis (RA), an autoimmune disease, is characterized by the presence of symmetric polyarthritis predominantly of the small joints that leads to severe cartilage and bone destruction. Based on animal and human data, the pathophysiology of osteoporosis, a frequent comorbidity in conjunction with RA, was delineated. Autoimmune inflammatory processes, which lead to a systemic upregulation of inflammatory and osteoclastogenic cytokines, the production of autoantibodies, and Th cell senescence with a presumed disability to control the systemic immune system’s and osteoclastogenic status, may play important roles in the pathophysiology of osteoporosis in RA. Consequently, osteoclast activity increases, osteoblast function decreases and bone metabolic and mechanical properties deteriorate. Although a number of disease-modifying drugs to treat joint inflammation are available, data on the ability of these drugs to prevent fragility fractures are limited. Thus, specific treatment of osteoporosis should be considered in patients with RA and an associated increased risk of fragility fractures.

## 1. Introduction

Rheumatoid arthritis (RA) is a classic autoimmune disease that affects approximately 1% of the population. The disease is characterized by symmetric polyarthritis predominantly of the small joints that leads to severe cartilage and bone damage [1,2]. Bone destruction in RA can occur either in the vicinity of inflamed joints or systemically. In this thematic context, Omata et al., described current aspects of the role of type 2 cytokines in regulating bone metabolism in inflammatory arthritides [3]; developing on the aforementioned work, we herein reviewed the molecular pathophysiology of systemic osteoporosis in RA.

Osteoporosis is defined as a “skeletal disorder characterized by compromised bone strength predisposing a person to an increased risk of fracture” [4]. Fragility fractures are associated with significant morbidity and mortality and frequently require the complex transdisciplinary management of patients [5]. In general, osteoporosis can be differentiated into primary and secondary forms. Examples of primary osteoporosis include postmenopausal and age-related osteoporosis; secondary forms of osteoporosis are caused by specific diseases or medications. Osteoporosis in conjunction with RA thus, in clinical praxis, is regarded as an example of secondary osteoporosis. A meta-analysis conducted by Jin et al., in patients with RA revealed an increased risk of fragility fractures (relative risk: 1.61) [6]. Vertebral fractures are the most frequent type of osteoporotic fractures; a recent meta-analysis found a 20% prevalence of vertebral fractures in RA patients, with the Odd’s ratio (OR) as high as 3.04 (confidence interval: 1.97–4.71) [7]. Bone fragility in RA results from both low bone mineral density (BMD) and from a severely deteriorated bone structure [8,9].

**Figure 1:**
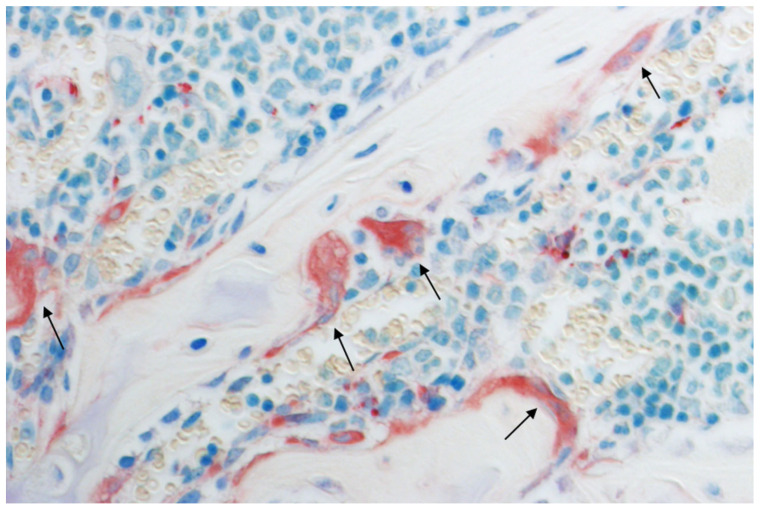
Multinucleated bone resorbing osteoclasts (black arrows), histological section of a mouse humerus stained with tartrate-resistant acid phosphatase (TRAP) and toluidine blue. Original magnification: 400×.

Osteoporosis is by far the most frequent metabolic bone disease and associated with a significant social and economic burden; it is regarded as a gender specific heterogeneous disorder that results from an imbalance of bone remodeling, i.e., the coupled processes of bone formation and bone resorption (Figure 1). For a detailed description of bone biology, bone remodeling and its regulation, the reader is referred to previous publications [10,11,12,13,14,15,16]. In this narrative review, we delineated the pathophysiology of osteoporosis in RA from different perspectives. First, we collected the evidence from veterinary medicine and animal models. In the second part, data on pathogenic factors in humans are presented. In the final part, results from intervention studies in humans are analyzed and clinical recommendations to optimize bone health in RA are provided. As far as possible we include results from meta-analyses.

## 2. Animal Studies

Rheumatoid arthritis is a very complex pathological condition similar to osteoporosis, but osteoporosis mainly affects the axial skeleton, whereas RA is restricted to the joints of the appendicular skeleton. So, although these two pathological entities primarily affect bone, both also have the characteristic of systemic diseases and are associated with an increased osteoclast activity and concomitantly elevated RANKL (receptor activator of nuclear factor κB ligand), the master osteoclastogenic cytokine, titers. However, the extent to which RA and osteoporosis are related to each other it is still not entirely clear. In other words, it is interesting to clarify whether patients suffering from RA also exhibit signs of osteoporosis, which would best be reflected in axial skeleton dysfunction. As a consequence, rodent models of RA are also expected to show signs of such an interrelation, if there is a connection between RA and osteoporosis, which they do [17,18]. Besides, RA is not found in large veterinary species such as in pigs in the way it is found in humans [19], although in dogs there exists a pathological condition very reminiscent of, but not identical to human RA, namely, **immune-mediated polyarthritis** (IMPA). In the erosive form, there is no association with gender or breed, whereas in the immune-mediated non-erosive form, mainly juvenile, male dogs of distinct DLA-DRB1 alleles are affected. Additionally, in the erosive form, no autoantigen is known; therefore, in summary the term RA should be avoided when speaking about the erosive form in dogs. As in human RA, the affected dogs exhibit signs of synovitis, pannus formation, infiltrating lymphocytes, neutrophils, and macrophages as well as an arteritis and glomerulonephritis on the systemic level. The erosive form is progressive and characterized by immune complex deposition in the ankylosing joints. Most probably due to molecular mimicry, affected dogs often possess antibodies against the canine distemper virus, whereas the presence of rheumatoid factors is not a reliable sign for erosive IMPA, as these are often found in dogs without any respective clinical signs. Mild, non-regenerative anemia can be diagnosed in a subpopulation of affected dogs and is indicative of the systemic character of the disease; however, contrary to RA, no signs of osteoporosis have been detected in IMPA so far [20].

The established rodent models of RA reflect the pathophysiological situation satisfactorily on the focal (including redness, joint swelling, cartilage, and bone destruction) as well as the systemic level with upregulated proinflammatory cytokines. These are adjuvant-induced arthritis, collagen-induced arthritis, and tumor necrosis factor-α (TNF-α) transgenic mice. 

Classically, **adjuvant-induced arthritis (AIA)** is induced by an intradermal injection of heat-killed *Mycobacterium tuberculosis* in paraffin oil at the base of the tail [21]. Usually, first clinical signs are observed nine days after the injection and present as hind paw swelling and locomotory difficulties. Alternatively, mice may be immunized intradermally with methylated BSA (mBSA) emulsified in complete Freund’s adjuvant [22]. Additionally, mice are intraperitoneally injected with *Bordetella pertussis*. The acute phase of AIA lasts for one week starting after the booster injection and is followed by a chronic phase.

In order to induce **collagen-induced arthritis (CIA)**, mice are subjected to an intradermal injection or intravenous infusion of heterologous type II collagen emulsified 1:1 with incomplete Freund’s adjuvant [23,24]. According to other regimens, CIA is provoked by means of intradermal immunization with collagen emulsified in complete Freund’s adjuvant, followed by a booster dose of collagen emulsified in incomplete Freund’s adjuvant three weeks later. As in humans, disease susceptibility is strongly linked to the MHC class II molecule, such as the DBA/1, B10.Q, and B10.III haplotypes. Only healthy, male mice at an age of approximately two months should be used as these develop the pathologic phenotype earlier than females. CIA is consistently induced 16–35 days after immunization in 90–100% of male mice and in 60–100% of females using bovine collagen. 

Interestingly, AIA and CIA are mediated by distinct immunopathogenic mechanisms, as AIA is driven mainly by TNF-α, whereas the key cytokine in CIA is interleukin (IL)-1 [23,24,25,26]. However, both models are characterized by a systemic upregulation of acute-phase proteins, IL-1β, IL-8, CCL2, and RANKL, whereas TNF-α, IL-17, and prostaglandin E_2_ are elevated exclusively in clinical AIA. Others found a higher ratio of systemic IL-17/IFN-γ in the CIA model [27]. Neutralization of interferon-γ (IFN-γ) accelerated the course of CIA and was associated with increased IL-17 levels in serum and joints, suggesting that the immune events leading to joint inflammation are a consequence of a disbalance of Th1, Th2, and Th17 cytokines.

More recently, the **serum transfer arthritis** model was generated, which is based on T cells expressing a single autoreactive TCR recognizing glucose-6-phosphate isomerase (G6PI). These cells escape negative selection in mice bearing a specific MHC class II allele, namely IAg7, and thus are responsible for a breach in B cell tolerance leading to high levels of anti-G6PI antibodies. These are the basis for a destructive and erosive arthritis similar to that seen in human RA. The adoptive transfer of serum from these mice results in peripheral joint swelling in most recipient strains. FcγRII^-/-^ mice exhibit an accelerated arthritis, whereas FcγRIII^-/-^ mice experience a more slowly developing arthritis. The K/BxN serum transfer model of arthritis shows an acute phase, which is modulated by FcγRII and FcγRIII, and a subacute phase, which results in bone erosion, even in the absence of FcγR signaling [28]. However, a literature search for osteoporotic signs in this model returned no positive results. On the other hand, **TNF-α transgenic mice** develop a chronic inflammatory and destructive polyarthritis within six weeks after birth, which is accompanied by trabecular and cortical bone loss as measured in the lumbar vertebrae and long bones [29,30,31].

The osteoporotic phenotype in these models is especially evident in CIA models. CIA rats exhibit decreased BMD in lumbar vertebrae and the femur, thus reflecting human osteoporosis to a high degree [32]. As in their human counterpart, not only the trabecular bone is severely affected, but also the cortical bone. In-depth analysis showed that not only did osteoclast activity increase but bone formation also decreased, which was underlined by increased RANKL and decreased dickkopf-1 (Dkk-1) expressions in the ankle joints. Besides CIA mice and rats, AIA models also point towards an association of RA and osteoporosis. In both models, RANKL was shown to be the main driver of BMD loss not only in the hook but also in the vertebrae, whereas the proinflammatory cytokines TNF-α and IL-1, which are among the prominent factors crucial for the pathogenesis of RA, seem not to contribute to the vertebral osteoporotic phenotype [21,24,33].

As is typical for osteoporosis, not only the vertebrae are affected in these models, but also the near-axial appendicular skeleton, that is primarily the proximal femur. This was demonstrated in another CIA study, that investigated the effects of the pan-JAK inhibitor peficitinib and the TNF-α-binding fusion protein etanercept on joint inflammation and BMD [34]. In this setting, peficitinib ameliorated arthritis and additionally improved BMD in the femoral metaphysis, which could not be achieved by etanercept. Additionally, RANKL production was suppressed by peficitinib, but not by etanercept, which is in line with the conclusions on the pathophysiology of the RA-related osteoporosis delineated from the studies mentioned before. Besides RANKL, also IL-6 seems to contribute to the osteoporotic phenotype of CIA rodents, as was demonstrated by inhibiting systemic IL-6 by an experimental antibody [35]. With this treatment, not only was BMD improved in the femur and lumbar vertebrae, but also bone structure and bone strength improved significantly.

## 3. Conclusions from Animal Studies

These models allow us to reach the conclusion that osteoporosis, at least in these experimental settings, is not primarily caused by inflammation, but mainly based upon an osteoimmune dysregulation characterized by an imbalance between osteoclast and osteoblast formation and activity through an up-regulation of RANKL. This conclusion is underlined by the fact that the osteoporotic phenotype in TNF-α transgenic mice cannot be combated with an anti-TNF-α treatment, but by sclerostin-inhibition, pointing out the importance of the RANKL–OPG balance in the pathophysiology of these models as well as in human osteoporosis.

## 4. Human Association Studies

Building upon the data from animal studies, in this part of the manuscript a concept of the pathophysiology of osteoporosis in patients with RA is developed.

It is well established that in RA **inflammatory cytokines** are responsible for localized bone alterations, e.g., bone erosions. Nevertheless, in RA several alterations in systemic cytokine titers can be detected, which may influence bone remodeling and, consequently, the risk of osteoporotic fractures. In a recently published study, Qiu et al., determined disease activity and systemic cytokine levels in RA patients with and without osteoporosis [36]. Serum levels of TNF-α, IL-6, and IL-17 were higher and levels of IL-4 and IL-10 were lower in RA patients when compared to those without osteoporosis. The authors also suggested that a combination of DAS28 (a disease activity score), IL-4, IL-10, and IL-17 could predict the incidence of osteoporosis in RA.

A study using flow cytometry to characterize peripheral blood lymphocyte subsets in RA patients demonstrated a higher **Th17:Treg ratio** in patients when compared to those without osteoporosis [37]. Interestingly, in the presence of osteoporosis the number of peripheral blood B cells was reduced. Verbruggen et al., analyzed monocyte cytokine production by flow cytometry in RA patients and reported a negative correlation between lumbar spine Z scores and the production of IL-1β and IL-6 [38]. Moreover, positive correlations of plasma concentrations of osteocalcin, a marker of bone formation and the production of IL1-β, IL-6, and TNF-α were observed. In line with the aforementioned study, Abdel Meguid and coworkers found a significant negative correlation of IL-6 levels with BMD in RA patients [39].

Caetano-Lopes et al., determined femoral bone gene expressions in patients with RA and compared the data with subjects diagnosed with primary osteoporosis [40]. Bone microarchitecture and mechanical bone properties in the RA patients and the patients with osteoporosis were similar. IL-17 (but not IL-1β, IL-6, and TNF-α) expression was significantly upregulated in RA; moreover, in RA an increased expression of Wnt10b and the Wnt-antagonist Dkk-1 was apparent. IL-17 thus appears to play a significant role in the bone pathophysiology of RA. 

The importance of specific circulating lymphocyte subsets for systemic bone loss in RA is further supported by studies on **senescent T cells**. CD28 is expressed by CD4^+^ cells and is important for T cell coactivation; CD4^+^ cells that are negative for CD28 are regarded as senescent. Fessler and coworkers showed that the frequency of CD4^+^CD28^-^ cells is significantly higher in RA patients with low BMD [41]. When CD4^+^ cells were isolated from RA patients, it was found that senescent CD4^+^ cells produced higher amounts of RANKL than CD4^+^CD28^+^ cells. This finding mechanistically links T cell senescence to osteoclast generation and, consequently, osteoporosis. Several studies in patients with RA reported negative correlations of (lumbar spine or hip) BMD with serum RANKL concentrations [41,42,43,44]. IL-37 is an anti-inflammatory cytokine; unexpectedly, significantly increased IL-37 levels were in patients with RA and osteopenia or osteoporosis [45]. 

The relation of **disease activity** to osteoporosis was investigated in a relatively high number of studies. Although neutral data were also published [46], the vast majority of reports found that in RA a higher disease activity is associated with bone loss and/or osteoporosis [47,48,49,50]. Indirectly, this association underlines the important role of the immunological disease process for systemic bone alterations in RA. Most studies on bone turnover markers found increased markers of bone resorption and decreased markers of bone formation; in particular, bone resorption correlated with disease activity [51,52].

**Anti-citrullinated protein antibodies** (ACPAs) are important autoantibodies involved in the pathogenesis of RA. They are used, alongside from the rheumatoid factor (RF), as diagnostic markers for RA [53]. Moreover, ACPAs are described to be relevant for prognosis, as they are linked to a more severe disease progression [54]. Nam et al., showed that ACPA-positive subjects with a new onset of musculoskeletal symptoms have a relative risk of 66.8% for developing RA [55]. Furthermore, ACPAs are assumed to mediate bone loss, as RA patients show a low bone mass at the beginning of the disease [53]. ACPAs are associated with systemic bone loss as they are able to bind to citrullinated vimentin on the osteoclast surface, causing differentiation and activation of these cells [54]. Kleyer et al., described that ACPA-positive patients show a significantly lower BMD in comparison to ACPA-negative subjects [56]. In a publication of Stemmler et al., similar observations were made. Furthermore, both papers described cortical and trabecular bone changes in ACPA-positive patients in the absence of clinical signs of arthritis [56,57]. Bugatti and coworkers showed that ACPA-positive individuals had significantly lower Z-scores in the lumbar spine [58]. Bruno et al., reported a connection between ACPA-positive patients and osteopenia and osteoporosis, respectively [59]. A publication of Cheng et al., demonstrated that RA-osteoporosis patients showed higher ACPA titers in comparison to non-osteoporotic RA patients [37]. Apart from ACPAs and RF, anti-carbamylated protein antibodies (anti-CarP) also appeared to be involved in enhancing bone resorption in RA patients [54]. Hauser et al., described that auto-antibodies against osteoprotegerin (OPG) are linked to elevated bone resorption in patients with RA [60]. Taken together, ACPAs and perhaps also other autoantibodies are associated with systemic bone loss in RA.

The aforementioned studies very consistently demonstrate that RA patients with and without systemic osteoporosis exhibit differences in systemic cytokine levels, peripheral blood lymphocyte subsets, and autoantibodies. Overall, the data suggest that osteoporosis in RA is strongly associated with inflammation. Nevertheless, in RA general risk factors for osteoporosis (such as sex, age, and treatment influences) may also impact bone turnover and, consequently, fracture risk. In the following paragraphs, these factors are discussed.

It is well established that **glucocorticoids** have detrimental effects on bone; glucocorticoid-induced osteoporosis generally is regarded as the most frequent secondary cause of osteoporosis [61]. In contrast to other autoimmune diseases (such as systemic lupus erythematosus), there is controversy concerning RA, concerning whether glucocorticoids (that inhibit inflammatory processes) indeed harm bone. Blavnsfeldt and coworkers reported a neutral effect of glucocorticoids on BMD in patients with early and active RA in a meta-analysis [62]. On the other hand, the meta-analysis of Wang et al., concluded that glucocorticoids in RA have unfavorable effects on bone [63]. As also discussed in the following paragraphs, high (but not low) doses of glucocorticoids in RA appear to increase the risk of osteoporosis.

In the pathogenesis of peripheral fragility fractures, in addition to osteoporosis, **sarcopenia** is also of relevance [64]. In a recently published study, BMD and skeletal muscle mass were assessed in 549 patients with RA and 158 control subjects. Both osteoporosis and sarcopenia were more prevalent in the RA patients than the controls; in patients with sarcopenia, a significantly higher incidence of osteoporosis was seen [65]. Logistic regression analyses revealed that female sex, sarcopenia, and advanced age were risk factors for osteoporosis in RA. 

With regard to the role of **general risk factors** of osteoporosis in RA, the literature is quite consistent. Ölzner et al., reported that osteoporosis was significantly more frequent in postmenopausal and male RA patients when compared to premenopausal patients [66]. In premenopausal women, low body mass index (BMI) was a risk factor of osteoporosis; whereas in postmenopausal women, alongside low BMI, advanced age and cumulative glucocorticoid doses were risk factors. In men with RA, low BMI and high cumulative doses of glucocorticoids were identified as osteoporosis risk factors. These findings are consistent with a study from Pakistan demonstrating that advanced age and lower BMI were associated with osteoporosis in postmenopausal women with RA [67]. In a study performed exclusively in male patients with RA, low BMI and disease activity (as assessed by DAS28-ESR) were risk factors of osteoporosis [68].

In women with RA and an age >50 years, vitamin D deficiency was significantly associated with new clinical and new osteoporotic fractures [69]. Hu et al., assessed risk factors for osteoporosis in 405 patients with RA and concluded that high serum levels of vitamin D protected against bone loss whereas advanced age, low BMI, and increased serum uric acid levels were associated with osteoporosis [70]. Overall, this part can be summarized such that the following general risk factors of osteoporosis are also effective in RA: post-menopause, advanced age, low body mass index, sarcopenia, vitamin D deficiency, and high cumulative doses of glucocorticoids.

## 5. Proof of Concept: Human Pharmacologic Intervention Studies

As stated above, systemic bone loss is of multifactorial origin but high levels of inflammatory cytokines are an important pathogenic factor. In RA patients with a Simplified Disease Activity Index (SDAI) above 3.3, fragility fracture risk is higher than in RA patients in remission (SDAI ≤ 3.3) [71]. In recent decades, disease-modifying antirheumatic drugs (DMARDs), especially biological/targeted synthetic DMARDs (b/tsDMARDs) directly targeting pathological cytokines were developed. According to the German National Database the frequency of osteoporosis decreased from 2007 (20%) until 2017 (7%) [72]. The decline in long-term glucocorticoid use and more effective disease control seem to be responsible for this positive development.

### 5.1. Bone Mineral Density

Favorable effects of **bDMARDs**—especially anti-TNF-α therapies—on bone metabolism and BMD have been shown (for review see [73,74,75,76]. After long-term treatment (three years), BMD remained stable in RA patients receiving different b/tsDMARDs, whereas it decreased in RA patients on conventional synthetic DMARDs (csDMARDs) [77]. The BMD of patients receiving the IL-6 receptor antibody tocilizumab remained stable or even improved slightly [78,79,80,81]. Concerning the protection of systematic bone loss, two studies point to the superiority of the CTLA4 antagonist abatacept compared to other bDMARDs [82,83]. Rituximab, a monoclonal antibody directed against the CD20 receptor on B cells, also seems to have a slight positive effect on bone health [84,85]. So far, only one clinical study evaluated the impact of janus kinase inhibition on bone health. A one-year treatment with tofacitinib stabilized BMD of the lumbar spine and hip region as well as volumetric BMD of the ultra-distal forearm [86]. From a pathophysiological point of view, the inhibition of IL-17 would definitely make sense in RA patients. However, no IL-17 inhibitors are approved for the treatment of RA.

**Denosumab**, a fully humanized monoclonal antibody against RANKL—initially developed for the treatment of osteoporosis—proved effective in RA patients, positively influencing erosions and BMD—even in patients concurrently receiving glucocorticoids—more than 10 years ago [87]. In Japan, denosumab is approved for the treatment of patients with RA. Two recent meta-analyses, one including 10 studies [88] and the other including 18 studies [89] showed increases in BMD of the lumbar spine (mean difference 5.12% and 4.07%, respectively) and total hip region (MD 2.82% and 2.43%, respectively). A comparison of TNF-α inhibitors, tocilizumab, or abatacept combined with denosumab suggested superiority of the IL-6 receptor antibody because of a significantly higher increase in hip BMD compared to the other regimens after 18 months [90].

Since DMARDs have a powerful anti-inflammatory activity and, thus, the potential to achieve clinical remission, one might suspect a low fracture incidence in RA patients on DMARDs. In the following part of the manuscript, we describe the effect of disease-modifying treatment on fragility fracture risk.

### 5.2. Non-Vertebral Fracture Risk

A subgroup analysis of the Women’s Health Initiative (WHI) study on postmenopausal women suffering from RA treated with **csDMARDs** detected that neither methotrexate, sulfasalazine, nor hydroxychloroquine had a substantial effect on fracture risk [91]. We would like to mention that methotrexate-related osteopathy (triad of pain, osteoporosis, and atypical fractures) is rare (for review see [92]). The longitudinal registration FORWARD showed that compared to MTX monotherapy, **TNF-α inhibition** and therapy with other synthetic DMARDs was not associated with a lower risk of non-vertebral fractures [93]. After multivariable adjustment for osteoporosis and fracture-related risk factors, the risk of non-vertebral osteoporotic fractures was not different between users of TNF-α inhibitors (HR 1.07) and csDMARDs [94]. A pooled analysis of nine studies evaluating all fracture types did not reveal a reduction in fracture risk in RA patients on bDMARDs (OR 1.07) either [95]. More than 130,000 RA patients initiating or switching to a b/tsDMARD were included in a large real-world study; it showed that the risk of non-vertebral fractures (hip, humerus, pelvis, and the wrist region) was similar between patients on adalinumab and those on any other TNF-α inhibitor, tocilizumab, abatacept, rituximab, or tofacitinib [96]. A recent meta-analysis, which included 37 RCTs (randomized controlled trials) comparing bDMARDs with placebo or csDMARDs, also did not detect a risk reduction in non-vertebral fractures, hip fractures, or major osteoporotic fractures [97]. Subgroup analyses of different bDMARDs did not change these findings. Not included in this meta-analysis was a recent prospective study following more than 4000 bDMARD (infliximab, adalimumab, etanercept, certolizumab, golimumab, abatacept, tocilizumab, rituximab, anakinra) users and matched bDMARD-naïve patients [98]. Abtahi and coworkers showed that the usage of bDMARDs is not associated with a difference in the occurrence of a first incident fragility fracture (including any osteoporotic fracture, hip fracture, clinical vertebral fracture, humerus fracture, forearm fracture). Similar results were obtained by a population-based cohort study, which reported no difference in non-vertebral fracture risk between patients treated with TNF-α inhibitors (infliximab, adalimumab, etanercept, or golimumab), the IL-6 receptor antibody tocilizumab, or the CTLA4 antagonist abatacept [99]. No data deriving from clinical studies conducted with the anti-CD 20 monoclonal antibody rituximab or JAK inhibitors investigating the effect on non-vertebral fracture risk exist. A retrospective analysis of RA patients receiving denosumab showed a decrease in clinical fractures (−49.2% after a mean of 4.6 denosumab applications) compared with the first six months of treatment as the reference period [100].

### 5.3. Vertebral Fracture Risk

Despite the high risk of vertebral fractures for RA patients (OR 3.04) [7], only a few studies evaluated the effect of different treatment regimens on vertebral fracture risk. A population-based retrospective cohort study showed that in RA patients with cardiovascular disease, low-dose corticosteroids (HR 0.57) as well as hydroxychloroquine (HR 0.12) lowered the risk of vertebral fractures [101]. Another retrospective cohort study, which followed RA patients for two years after the initiation of a TNF-α antagonist, detected a risk reduction of clinical vertebral fractures (HR 0.71, adjusted for baseline glucocorticoid use); the references were users of csDMARDs [102]. The extent of vertebral fracture risk reduction was similar in a longitudinal observational study, which enrolled more than 11.000 RA patients without prior fracture. Compared to MTX monotherapy, TNF-α inhibition was associated with a lower risk of vertebral fractures (HR 0.72) [93]. In their analysis of RA patients with cardiovascular disease, Hong and coauthors [101] showed a non-significant reduction of the risk of osteoporotic vertebral fractures induced by the application of TNF-α inhibitors. A meta-analysis revealed a reduction of vertebral fracture risk (OR 0.71) for patients receiving bDMARDs compared with bDMARDs non-users [95]. So far, no studies evaluating the effectiveness of other b/tsDMARDs concerning vertebral fracture risk reduction have been published.

### 5.4. Fracture Healing

Despite the high number of disease-modifying drugs and the relatively good control of disease activity nowadays, fragility fractures occur in many RA patients. What about fracture healing? Available data deriving from experimental studies conducted with mice and rats have demonstrated that anti-TNF-α treatment does not interfere with fracture healing [103] whereas with MTX only the low dose group did not show a difference in bone formation compared with a control group [104]. After a hip fracture, readmission rate and mortality seem to be similar in patients on bDMARDs and methotrexate [105]. Based on their in vitro results, which showed the promotion of bone formation, Gaber et al., hypothesized that in case of a fracture patients do not have to stop tofacitinib treatment [106].

### 5.5. Sarcopenia

In general, aiming at fracture prevention we mainly focused on bone density. Besides this very important approach, however, we should keep rheumatoid cachexia and the risk of falling in mind as well. The pathogenesis of sarcopenia/falls in RA is incompletely understood; important determinants are disease activity, pain, reduced mobility, fatigue and glucocorticoid treatment [107]. The one-year inhibition of IL-6 by tocilizumab increased lean mass in RA patients [108] and the use of bDMARDs seems to divide the risk of sarcopenia by half (OR 0.51) [109]. The direct effects of cytokine inhibition or indirect effects as described before could be responsible for this effect.

## 6. Non-Pharmacologic Intervention Studies

As mentioned above, vitamin D deficiency in RA is detrimental for bone health. Since vitamin D in addition to its skeletal effects also exerts immunomodulatory actions, the possibility exists that vitamin D supplementation in RA could influence disease activity. In fact, a meta-analysis conducted by Guan et al., reported that vitamin D improved DAS28, erythrocyte sedimentation rate, and tender joint count [110]. In contrast, a meta-analysis conducted by Nguyen et al., concluded that effects of vitamin D supplementation on disease activity in RA were limited [111]. Long term exercise interventions in RA were shown to slow down the decline of BMD in RA [112]. A decrease in disease activity (e.g., by pharmacologic interventions) likely will result in improved mobility and potentially in increased sun exposure (and consequently higher vitamin D levels) and positive effects on BMD. Thus, there are complex interactions between pharmacologic and non-pharmacologic pathways.

## 7. Conclusions

**Figure 2:**
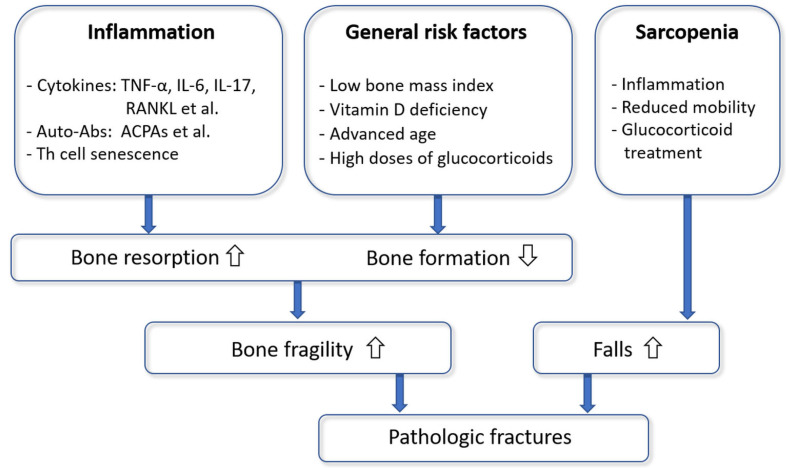
A model of the pathophysiology of osteoporotic fractures in rheumatoid arthritis.

In summary, we proposed the following model of the pathophysiology of osteoporosis in RA (Figure 2). The autoimmune inflammatory processes lead to a systemic upregulation of inflammatory and osteoclastogenic cytokines (in particular RANKL and TNF-α), the production of autoantibodies and Th cell senescence with a presumed disability to control the systemic immunological and osteoclastogenic status. Consequently, osteoclast activity increases, osteoblast function decreases, and bone metabolic and mechanical properties deteriorate. General risk factors of osteoporosis (such as advanced age or vitamin D deficiency) may further contribute to bone fragility. Sarcopenia in RA is a cause of falls and their potential consequence, fractures.

The high number of disease-modifying drugs increases our ability to achieve good control of disease activity. However, the effects of these drugs on fragility fracture risk are limited. The most promising evidence exists for TNF-α inhibitors, which proved the reduction of vertebral fracture risk in a meta-analysis. Positive data derived from clinical studies conducted with denosumab exist. In contrast, DMARD treatment is not sufficient for RA patients prone to osteoporotic fractures. In these patients, a bone-specific medication, accompanied by sufficient calcium intake and serum levels of vitamin D within the desirable range, is necessary. Further progress in osteoimmune research will help to develop new treatment options in RA-osteoporosis patients [113].

We thus recommend a holistic “treat to target” approach for the optimal management of RA patients. Patient-centered care should include adequate control of disease activity, osteoporosis prevention/treatment, and regular physical exercises aiming at increased muscle strength and/or fall prevention depending on the patients’ needs.

## Data Availability

Not applicable.
